# Organic Anion Transporting Polypeptide 2B1 in Human Fetal Membranes: A Novel Gatekeeper for Drug Transport During Pregnancy?

**DOI:** 10.3389/fphar.2021.771818

**Published:** 2021-12-20

**Authors:** Esha Ganguly, Ananth Kumar Kammala, Meagan Benson, Lauren S. Richardson, Arum Han, Ramkumar Menon

**Affiliations:** ^1^ Division of Basic and Translational Research, Department of Obstetrics and Gynecology, The University of Texas Medical Branch at Galveston, Galveston, TX, United States; ^2^ Department of Electrical and Computer Engineering, Texas A&M University, College Station, TX, United States

**Keywords:** transporter protein, drug transport, pregnancy, human fetal membranes, organ-on-a-chip

## Abstract

Current intervention strategies have not been successful in reducing the risks of adverse pregnancy complications nor maternal and fetal morbidities associated with pregnancy complications. Improving pregnancy and neonatal outcomes requires a better understanding of drug transport mechanisms at the feto-maternal interfaces, specifically the placenta and fetal membrane (FM). The role of several solute carrier uptake transporter proteins (TPs), such as the organic anion transporting polypeptide 2B1 (OATP2B1) in transporting drug across the placenta, is well-established. However, the mechanistic role of FMs in this drug transport has not yet been elucidated. We hypothesize that human FMs express OATP2B1 and functions as an alternate gatekeeper for drug transport at the feto-maternal interface. We determined the expression of OATP2B1 in term, not-in-labor, FM tissues and human FM cells [amnion epithelial cell (AEC), chorion trophoblast cell (CTC), and mesenchymal cells] using western blot analyses and their localization using immunohistochemistry. Changes in OATP2B1 expression was determined for up to 48 h after stimulation with cigarette smoke extract (CSE), an inducer of oxidative stress. The functional role of OATP2B1 was determined by flow cytometry using a zombie violet dye substrate assay. After OATP2B1 gene silencing, its functional relevance in drug transport through the feto-maternal interface was tested using a recently developed feto-maternal interface organ-on-a-chip (OOC) system that contained both FM and maternal decidual cells. Propagation of a drug (Rosuvastatin, that can be transported by OATP2B1) within the feto-maternal interface OOC system was determined by mass spectrometry. FMs express OATP2B1 in the CTC and AEC layers. In FM explants, OATP2B1 expression was not impacted by oxidative stress. Uptake of the zombie violet dye within AECs and CTCs showed OATP2B1 is functionally active. Silencing OATP2B1 in CTCs reduced Rosuvastatin propagation from the decidua to the fetal AEC layer within the feto-maternal interface-OOC model. Our data suggest that TPs in FMs may function as a drug transport system at the feto-maternal interface, a function that was previously thought to be performed exclusively by the placenta. This new knowledge will help improve drug delivery testing during pregnancy and contribute to designing drug delivery strategies to treat adverse pregnancy outcomes.

## Introduction

More than one in six pregnancies are affected by various pregnancy-related complications, including preterm birth, fetal growth restrictions, or preeclampsia, which are associated with substantial maternal, fetal, and neonatal morbidity and mortality (Collier and Molina, 2019). Despite the rising trend in pregnancy-related complications within the past decades in the United States, current treatment strategies have not been successful in reducing adverse pregnancy outcomes (D’Alton et al., 2019). Moreover, the current lack of knowledge about the effect of maternally administered drugs on the developing fetus is a major public healthcare concern worldwide (Sachdeva et al., 2009).

Unfortunately, and justifiably, concerns about fetal safety have limited pharmacotherapy and drug studies during pregnancy (Costantine, 2014). For example, maternal treatment with antiepileptic drugs results in fetal teratogenicity, such as intrauterine growth restriction, congenital anomalies, and infant mortality (Güveli et al., 2017). Given that therapy during pregnancy-related complications may expose the fetus to adverse effects *in utero*, a proper understanding of the passage of drugs across the feto-maternal interface will guide the development of more accurate and safer pharmacotherapy during pregnancy. The feto-maternal interface is comprised of the placenta-decidua basalis interface and the fetal membrane (FM) (i.e., amniochorion)-decidua parietalis interface, both acting as protective barriers throughout pregnancy while maintaining communication between the mother and the fetus (Menon et al., 2021). Disruption of these interfaces leads to various pregnancy complications, including preterm birth, which can have long-term adverse effects on maternal and fetal health (Menon et al., 2019). Thus, understanding the role of the feto-maternal interfaces in drug transport is critical to advancing clinical treatment strategies during pregnancy.

Drug transporter proteins play an essential role in regulating the transport of drugs across a variety of tissues such as intestinal enterocytes, kidneys (proximal tubule), hepatocytes, and brain (International Transporter et al., 2010). The distribution, substrate specificity, and activities of the membrane transporters are essential determinants of drug absorption, excretion, and in many cases, the extent of drug entry into target organs (Al-Enazy et al., 2017). The placenta-decidua basalis interface is the only region studied for drug transportation functions during pregnancy due to its direct interaction with maternal blood (Dallmann et al., 2019). Gestational age-dependent expression of transporter proteins in the placenta is crucial in limiting fetal toxicities by preventing entry or facilitating the active efflux of endogenous waste products and xenobiotics out of the fetal compartment (Anoshchenko et al., 2020). For example, ATP-Binding Cassette (ABC) efflux transporters such as breast cancer resistance protein (BCRP; ABCG2) and P-glycoprotein (P-gp; ABCB1) are among one of the highly expressed transporters in the placenta, and can transport diverse therapeutic drugs (such as antihypertensive, antivirals, and antibiotics), thus protecting the fetus from the effects of the treatment (Staud et al., 2012; Han et al., 2018). In addition to the ABC efflux transporters, the organic anionic transporters (OATs) and organic anion transporting polypeptides (OATPs) also play a critical role in the transport of various endogenous molecules and xenobiotics (Staud et al., 2012). OATs and OATPs mediate the transport of steroid sulfates, thyroid hormones, and drugs (e.g., statins), as well as the transport of waste products (Roth et al., 2012). An essential member of the OATP family is OATP2B1. In human placenta, OATP2B1 is expressed on the basal membrane of the syncytiotrophoblast (Grube et al., 2007). However, the physiological and pharmacological role of OATP2B1 is poorly understood, which is one of the focus of our study.

Despite being structurally and functionally different from the placental interface, the role of FM-decidua parietalis interface in drug transportation and function remains unclear. Of note, drugs have to pass through both placenta-decidua basalis and the FM-decidua parietalis to reach the fetus. Thus, it is important to understand whether drugs can indeed pass through the FM-decidua parietalis interface to reach the fetus. An ideal strategy will be the simultaneous testing of drug transporter expression and function at both interfaces to have a complete understanding of drug kinetics (i.e., absorption, distribution, metabolism, elimination) and its associated effects on the fetus. We hypothesize that human FMs express transporter proteins, such as OATP2B, which function as an alternate gatekeeper for drug transport at the feto-maternal interface.

## Methods

### IRB Ethics Committee Approval Statement

Placental samples used for this study were collected after normal term cesarean deliveries from John Sealy hospital [University of Texas Medical Branch (UTMB)] at Galveston, Texas, United States in accordance with the relevant guidelines and regulations of approved protocols for various studies (UTMB 11-251). As discarded placentas after delivery were used for the study, no subject recruitment or consent was required.

### Placental and FM Explant Culture

Placental and FM (i.e., amniochorion) tissues from term, not in labor, cesarean deliveries (TNIL) (*n* = 5) were used for this project and prepared as previously described by our laboratory (*n* = 6 tissues for western blotting and *n* = 5 tissues for explant culture) (Ayad et al., 2018; Sheller-Miller et al., 2020). Briefly, the FM was dissected from the placenta, washed three times in normal saline, cleaned with sterile saline-moistened gauze to clear any adherent blood clots and all the decidua, and washed in Hank’s balanced solution (HBSS) with penicillin 100 U/mL and streptomycin 100 µg/ml. Using a skin biopsy punch, the membranes were cut into 6-mm disks. Sections were taken from midzone of the fetal membranes, avoiding the regions overlying the cervix or placenta. Four disks of the amniochorion membranes were placed in each well of a 24-well tissue culture plate with 0.4 ml of Dulbecco’s modified Eagle’s medium (DMEM) with Ham F12 nutrient mixture 1:1; antibiotics: penicillin (100 IU/ml), streptomycin (100 µg/ml), and amphotericin B (2.5 µg/ml); glutamine (2 mmol/L); and 15% heat-inactivated fetal bovine serum. Cultures were incubated at 37°C with 5% CO_2_. The medium was changed at 24 h (hrs.) when the study reagents were added.

Placental explants were prepared as described previously (Behnia et al., 2016; Arita et al., 2018). Briefly, placental tissues were washed with sterile saline. After removal of the blood clots by gentle blotting, segments of the maternal part of the placental villi were isolated by sharp dissection, and subsequently washed and cut 3–5 times. Chopped tissues were washed in DMEM supplemented with 5% fetal bovine serum (v/v), penicillin (100 U/mL), and streptomycin (100 µg/ml). Placental tissue (0.1 gm) was placed in each well of a 24-well tissue culture plate in 0.5 ml DMEM with Ham F12 nutrient mixture 1:1; antibiotics: penicillin (100 IU/ml), streptomycin (100 µg/ml), and amphotericin B (2.5 µg/ml); glutamine (2 mmol/L); and 10% heat-inactivated fetal bovine serum. The medium was changed at 24 h when the study reagents were added.

### Culture of Immortalized Human FM Cells

Maternal decidual and FM cells [amnion epithelial cells (AECs); amnion mesenchymal cells (AMCs), chorionic trophoblast cells (CTCs)] were isolated from term, not-in-labor, cesarean-delivered FM and immortalized by a standard HPV16 E6E7 retrovirus protocol (Halbert et al., 1991). Retroviruses were obtained from the PA317 LXSN 16E6E7 (ATCC® CRL-2203™) cell line. Cells derived from healthy FM tissues were seeded in 6-well plates. The next day, cells were infected with 500 μL of the mixture containing 50 μL of retrovirus supernatant, 5 μg of protamine sulfate in serum-free culture medium, and incubated at 37°C for 6 h. Culture medium was replaced with fresh media, and 48 h after the viral infection, antibiotic selection was started. Five days after the antibiotic selection process, the immortalized cells were expanded and passaged several times (up to passage 8) before using them in the experiments. AMCs and maternal decidual cells were cultured and maintained in complete media consisting of Dulbecco’s modified Eagle’s medium (DMEM) with Ham F12 nutrient mixture 1:1; antibiotics: penicillin (100 IU/ml), streptomycin (100 µg/ml), and amphotericin B (2.5 µg/ml); and 5% heat-inactivated fetal bovine serum. AECs were cultured in complete keratinocyte serum-free media (KSFM), a culture highly selective for epithelial cells, supplemented with human recombinant epidermal growth factor (0.1 ng/ml), bovine pituitary extract (30 µg/ml), and primocin (0.5 mg/ml) (ant-pm-1; Invivogen). Supplements were added to the media immediately before use. CTCs were cultured in Dulbecco’s modified Eagle’s medium (DMEM) with Ham F12 nutrient mixture 1:1 supplemented with the following: fetal bovine serum, penicillin, streptomycin, bovine serum albumin, ITS-X, CHIR99021, A83-01, SB431542, L-ascorbic acid, epidermal growth factor, VPA, and Y27632 (ROCK inhibitor). Cells used for all experiments were within 10 passages.

### Stimulation of FM and Placental Explant Culture With Cigarette Smoke Extract

Cigarette smoke extract (CSE) was prepared as previously reported by our laboratory (Menon et al., 2013; Sheller et al., 2016; Richardson et al., 2020a). Briefly, smoke drawn from a single lit commercial cigarette (unfiltered Camel™, R.J. Reynolds Tobacco Co.; Winston Salem, NC) was bubbled through 50 ml of tissue culture medium (DMEM: F12 Ham’s mixture with antimicrobial agents and FBS). Each cigarette is reported to contain 26 mg of tar and 1.7 mg of nicotine. The stock CSE was filter-sterilized using a 0.25 µm pore Millipore filter (Millipore, Bedford, MA) to remove insoluble particles. Fetal membrane and placental explants were then stimulated with CSE (diluted 1:10 in culture media) for 0, 4, 8, 24 and 48 h. CSE was prepared freshly for each experiment. After the treatments, the explants (placental and FM) were removed and processed for western blotting for OATP2B1. Both placental and FM explants were used to better understand the expression levels of OATP2B1 in response to CSE stimulation.

### Immunohistochemistry for OATP2B1

Immunohistochemistry (IHC) for OATP2B1 was performed on FM tissues using methods previously described by our laboratory (Kammala et al., 2020). Briefly, FM sections were fixed in 4% paraformaldehyde for 48 h and embedded in paraffin. Tissue sections were cut to 5 μm thickness, placed on a positively charged slide, and attached by keeping them at 50°C for 45 min. Slides were deparaffinized using xylene, and then rehydrated with 100% alcohol, 95% alcohol, and normal saline (pH 7.4), followed by staining. Tissue sections were probed with the antihuman OATP2B1 antibody (1:500, ab222094, Abcam, Cambridge, United Kingdom) overnight at 4°C, and IHC anti-rabbit/anti-mouse secondary antibody (Mouse and Rabbit Specific HRP/DAB (ABC) Detection IHC kit, ab64264, Abcam, Cambridge, United Kingdom) was added for 45 min at room temperature, followed by adding DAB as a chromogen for 15 s, and then hematoxylin as a counterstain for color development. Bright-field microscopy images were captured using a Nikon Eclipse TS100 microscope (20x and 100x) (Nikon).

### Flow Cytometry for Surface Expression of Fluorophore Conjugated OATP2B1 in FM Cells

Firstly, anti-human OATP2B1 antibody (ab222094, Abcam, Cambridge, United Kingdom) was labeled using the CF® Dye and Biotin Protein Labeling Kits (Biotium, Fremont, CA) along with Rabbit IgG (AB_2532938, Invitrogen) as per the manufacturer’s protocol. The next day, the conjugated OATP2B1 antibody was used for flow cytometry analysis. Briefly, FM cells (AEC, AMC, and CTC), maternal decidua, and BeWo cells (used as a surrogate for placental cells) were harvested, washed in ice-cold PBS, 10% FCS, 1% sodium azide and centrifuged at 3,000 rpm for 10 min. Cell pellets were incubated with specific fluorophore conjugated antihuman OATP2B1 antibody (1:50) and propidium iodide dye (BioLegend, San Diego, CA) for 30 min at 4°C in dark. Cells were washed three times by centrifugation at 400 g for 5 min and resuspended in 500 µL of ice-cold PBS, 10% FCS, 1% sodium azide, and analyzed immediately on a CytoFLEX flow cytometer (Beckman Coulter, Brea, CA).

### Fluorescent Zombie Violet Dye Uptake Determined by Flow Cytometry in FM Cells

Based on our IHC and western blotting data, we chose AECs and CTCs to further study the uptake function of cell surface OATP2B1 by flow cytometry. Previous studies have demonstrated that intracellular accumulation of zombie violet dye is increased explicitly by OATPs (Patik et al., 2015; Patik et al., 2018). OATP2B1 is known to function almost exclusively at acidic extracellular pH (Patik et al., 2018). Based on these criteria, we chose the zombie violet dye uptake assay to determine the functional capacity of OATP2B1 in FM cells. Briefly, cells were washed in uptake buffer (125 mM NaCl, 4.8 mM KCl, 1.2 mM CaCl_2_, 1.2 mM KH_2_PO_4_, 12 mM MgSO_4_, 25 mM MES, and 5.6 mM glucose, with the pH adjusted to 5.5 using 1 M HEPES and 1 N NaOH). After washing 5 × 10^5^ cells were incubated for 15 min at 37°C with Zombie violet (BioLegend®, San Diego, CA, United States; 0.4 µL zombie violet/5 × 10^5^ cell) and propidium iodide dye (BioLegend, San Diego, CA) in a final volume of 100 µL. The reaction was stopped by adding 1 ml ice-cold PBS and the fluorescence of 10,000 living cells was immediately determined on a CytoFLEX flow cytometer (Beckman Coulter, Brea, CA). Dead cells were excluded by propidium iodide labeling.

### Protein Extraction and Western Blotting

Placental and FM explants as well as immortalized maternal DECs and FM cells (AECs, AMCs, CTCs) were lysed with radioimmunoprecipitation lysis buffer [50 mM Tris (pH 8.0), 150 mM NaCl, 1% Triton X-100, 1.0 mM EDTA (pH 8.0), and 0.1% SDS] supplemented with a protease and phosphatase inhibitor cocktail and also phenyl methyl sulfonyl fluoride. Human FM were homogenized as previously described (Richardson et al., 2020a). After centrifugation at 12,000 g for 20 min, the supernatant was collected and protein concentrations were determined using the Pierce bicinchoninic acid kit (Thermo Scientific, Waltham, MA, United States). The protein samples were separated using SDS-polyacrylamide gel electrophoresis on a gradient (4–15%) Mini-PROTEAN TGX Precast Gel (Bio-Rad, Hercules, CA, United States) and transferred to the membrane using a Bio-Rad Gel Transfer Device (Bio-Rad). Membranes were blocked in 5% non-fat milk in 1× Tris-buffered saline-Tween 20 for a minimum of 1 h at room temperature and then probed overnight at 4°C using primary antibody overnight. The membranes were then incubated with secondary antibody conjugated with horseradish peroxidase, and immunoreactive proteins were visualized using chemiluminescence reagents ECL WB detection system (Amersham Piscataway, NJ, United States). The following anti-human antibodies were used for western blot: OATP2B1 (1:1,000 for tissues and 1:250 for FM cells, ab245246, Abcam, United States) and β-actin (1:15,000, Sigma- Aldrich, St. Louis, MO, United States). Protein band quantification were normalized to β-actin expression in each lane, and the expression levels were densitometrically determined using the Bio-Rad Image Lab 6.0 software.

### Short-Interfering RNA-Mediated Gene Silencing of OATP2B1 in CTCs

CTCs were found to have maximum levels of OATP2B1 compared to all other cell types in FMs. Therefore, we chose CTCs to further study the role of OATP2B1 in the uptake of fluorescent zombie violet dye and substrate (Rosuvastatin) propagation across the FM layers based on our immunohistochemistry western blot analyses. CTCs were cultured to 50-60% confluency in DMEM/F12 medium supplemented with 10% FBS and antimicrobial agents (penicillin, streptomycin, and amphotericin), and then transfected with 15 µL of 20 µM oligos (Dharmacon, Lafayette, CO, United States) directed against OATP2B1 or nontarget scrambled (control) siRNAs using RNAimax lipofectamine as per the manufacturer’s instructions (Thermo Fisher Scientific, Waltham, MA, United States). After 24 h, OATP2B1 knockdown CTCs were separately used for the fluorescent zombie violet uptake assay BeWo cells was used as a surrogate of placental cells. BeWo cells is a commonly used *in vitro* model for studying the placental barrier. BeWo cells emulate a term human placental barrier in culture conditions because of its ability to fuse and form a syncytium *in vitro*. In another set of experiments, control and knockdown CTCs were loaded into the feto-maternal interface OOC device as previously described (Radnaa et al., 2021), as further described in the following section.

### Use of the Four-co-culture-chamber Feto-Maternal Interface OOC Device to Monitor the Propagation of Rosuvastatin Across Fetal Membrane Cells

The development and validation of the feto-maternal interface -OOC device have been previously reported by our laboratory (Richardson et al., 2019; Richardson et al., 2020b; Richardson et al., 2020c; Radnaa et al., 2021). In brief, the microfluidic feto-maternal interface -OOC is composed of four concentric cell culture compartments (one for maternal cells and three for fetal cells) interconnected through arrays of microfluidic channels to allow co-cultivation of four different cell types. Chamber one, the central chamber is for maternal DECs, chamber two is for fetal CTCs, chamber three is for AMCs, and the outermost chamber is for AECs. Each cell chamber is 250 μm in height, and the width of each chamber was designed to mimic the differences in thickness of each maternal and fetal layer as seen *in utero*. The chambers are interconnected through an array of 24 microchannels (all width: 35 μm; length: 600 µm for CTCs to DECs and AMCs to CTCs, 300 µm for AECs to AMCs; all height: 5 µm) that performed several functions, such as preventing the flow of cells between compartments during the initial cell loading process, allowing independent localized biochemical treatments to each compartment, enabling independent elution of supernatant from each cell compartment, and allowing biochemicals to diffuse between the chambers to enable cell-cell communication. The number of microchannels, along with the height and width, remained same throughout all experiments as to not affect the diffusion rate of molecules. The device contains an on-chip reservoir block placed on top of the cell culture chamber layer and is comprised of multiple 4 mm diameter and 2 mm deep reservoirs, where each reservoir is aligned in such a way that they are placed on top of the inlets and outlets of each cell culture chamber in the main cell culture layer. The center culture chamber has one reservoir on top of it and the outermost culture chamber has four reservoirs on top of it, while the two middle culture chambers have two reservoirs each on top of them, to provide sufficient cell culture medium to all parts of the cell culture chambers evenly. The feto-maternal interface -OOC was fabricated in polydimethyl siloxane (PDMS, 1:10 mixture, Sylgard 184; DowDuPont, Midland, MI, United States) using a two-step photolithography master mold fabrication process, followed by a soft lithography process of replica-molding the final PDMS device from the master mold (Park et al., 2010). The bonding of the PDMS layer onto the glass substrate was improved by treating the PDMS layers with oxygen plasma (Harrick Plasma, Ithaca, NY, United States), followed by bonding the layer onto a glass substrate. This process was repeated to bond the PDMS reservoir layer on top of the device. The feto-maternal interface -OOC device was stored dry (for up to 1 month) and before using the feto-maternal interface -OOC, the devices were sterilized with 70% ethanol for 15 min.

### Type IV Collagen Loading Into the Feto-Maternal Interface-OOC Microchannels

Before using the feto-maternal interface-OOC, the devices were washed three times with PBS, filled with type IV basement membrane collagen Matrigel (Corning Matrigel basement membrane matrix, DEV-free; 1:25 in media), and incubated at 37°C with 5% CO_2_ overnight. Diluted type IV collagen basement membrane Matrigel was used to fill the microfluidic channels between the AMC and the CTC compartments, mimicking the amnion and chorion basement membranes *in utero*. These Matrigel-filled microchannels will allow localized drug treatment of each cell layer and independently take supernatant from each layer. These channels also allow biochemical to diffuse between the layers and permit cell migration and transition, as seen in the fetal membrane-decidua feto-maternal interface.

### Cell Seeding and Culture in the Feto-Maternal Interface-OOC Device

After Matrigel loading, the cell chambers were rinsed two times with PBS to remove extra Matrigel, and the devices were loaded with immortalized cells in the different culture chambers. Immortalized cells were then trypsinized and loaded into the device, starting from the center chamber to the outside. Cell loading concentration in each chamber mimicked those of *in utero* cell ratios of the FM tissue (60,000 DECs for chamber one, 200,000 CTCs + 5% primary collagen + 25% Matrigel for chamber two, 62,500 AMCs + 20% primary collagen + 25% Matrigel for chamber three, and 120,000 AECs for chamber four). Following cell seeding, both the feto-maternal interface-OOC devices and the 24-well plates were incubated at 37°C with 5% CO_2_ overnight before Rosuvastatin treatment.

### Rosuvastatin Treatment Preparation

Rosuvastatin was obtained from Sigma-Aldrich (St Louis, MO). Rosuvastatin calcium salt was prepared as a stock of 10 mg/ml in serum-free media, and was added in a concentration of 200 nmol/L. The dose of rosuvastatin was chosen based on the maximum concentrations achievable in tissues after therapeutic doses, as previously reported by our laboratory (Ayad et al., 2018). Next day, rosuvastatin (200 ng/ml) was added to the maternal DEC chamber, and the supernatant from each chamber (AEC, AMC, CTC, and maternal DEC) was collected after 4 h. We chose this time point because previous studies from our laboratory have observed propagation of rosuvastatin from the maternal decidual chamber to the AEC chamber of the feto-maternal interface-OOC within 4 h after introducing the drug (unpublished data). To confirm the kinetics of Rosuvastatin in OATP2B1 knockdown CTCs, the concentration of rosuvastatin in the media collected from each of the chamber was analyzed by mass spectrometry (see below for details).

### Mass Spectrometry for Analysis of Rosuvastatin Propagation in the Feto-Maternal Interface -OOC

Targeted liquid chromatography tandem mass spectrometry (LC-QQQ) analysis was performed on a TSQ Altis mass spectrometer (Thermo Scientific, Waltham, MA) coupled to a binary pump UHPLC (Vanquish, Thermo Scientific). Scan parameters for target ions as per the Selective Reaction Monitoring (SRM Table) for Rosuvastatin were used. The injection volume was 10 µL. Chromatographic separation was achieved on a Hypersil GOLD™ C18 HPLC column (Dimensions: Particle size: 5 μm; Diameter: 50 mm; and Length: 2.1 mm) (Thermo Scientific) maintained at 30°C using a solvent gradient method. Solvent A was 0.1% formic acid in water. Solvent B was 0.1% formic acid in acetonitrile. The gradient method used was 0–1 min (20% B to 60% B), 1-2 min (60% B to 95% B), 2-4 min (95% B), 4–4.1 min (95% B to 20% B), and 4.1–5 min (20% B). The flow rate was 0.5 ml min^−1^. Sample acquisition and data analysis was performed using the Trace Finder 4.1 software (Thermo Scientific).

### Statistical Analysis

Statistical analysis and significance were determined by using the Prism 9 software (Graph Pad, San Diego, CA). Statistical analyses for normally distributed data were performed using analysis of variance (ANOVA) with the Tukey Multiple Comparisons Test. Significance is shown as *p < 0.05 and **p < 0.01. All data are shown as mean ± standard error of the mean (SEM).

## Results

### OATP2B1 Localization in FMs

**FIGURE 1:**
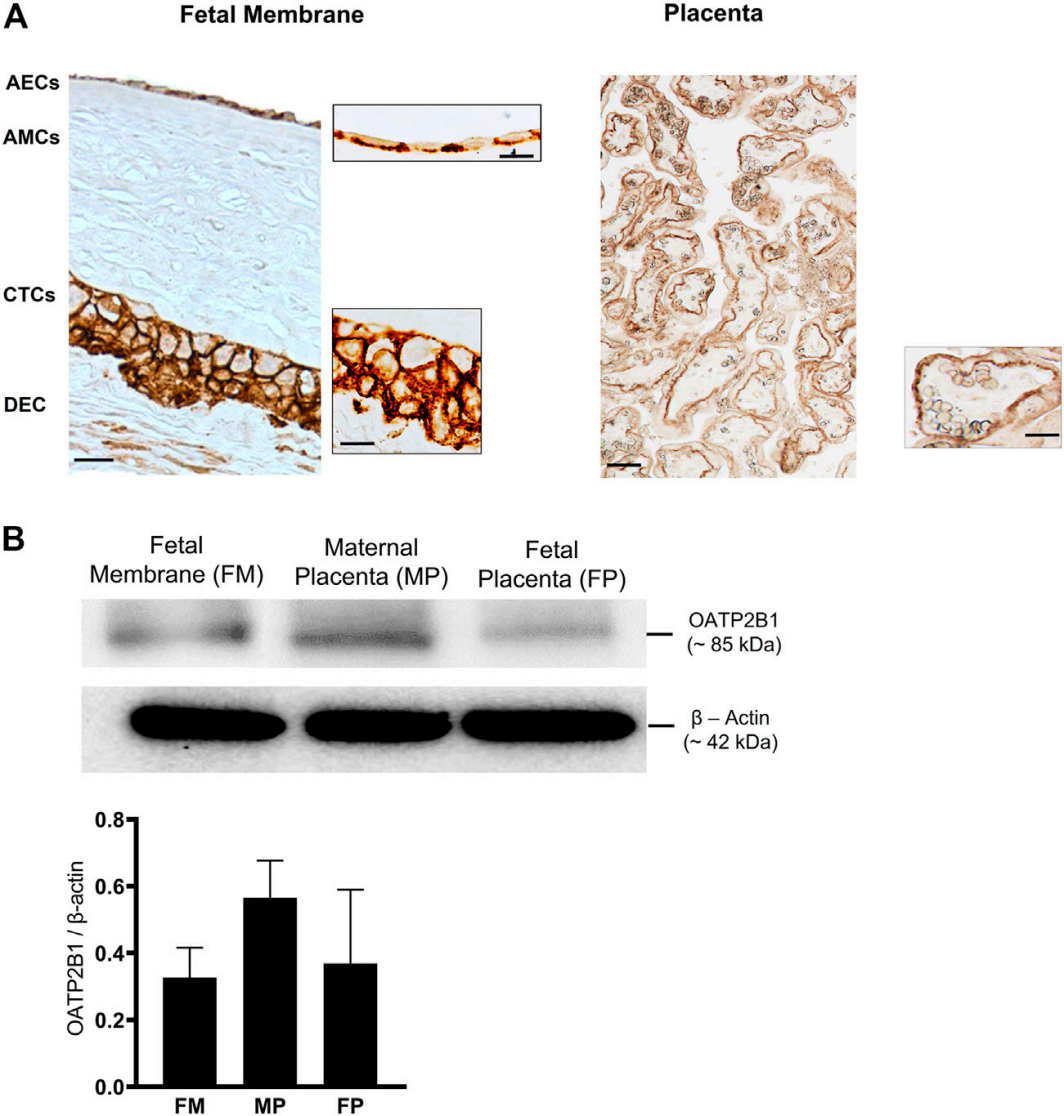
Localization and expression of OATP2B1 transporter protein in fetal membrane tissues and human placenta. **(A)** Bright field microscopy showing localization of OATP2B1 (brown) in human fetal membrane AECs and CTCs (left image) at a magnification of 20x (main image) and 100x (inset image) and placental syncytiotrophoblast at a magnification of 20x (main and inset image (right image). 20x and 100x images were taken in different regions of the tissue. Images are representative of three biological replicates. Scale bar = 30 µM. **(B)** Western blot analysis and quantification of OATP2B1 in human fetal membranes and placenta (maternal and fetal side). β-Actin is used as a loading control. Representative western blots are shown. *n* = 6 biological replicates. Error bars represent mean ±SEM.

FMs are divided into morphologically distinct amnion and chorion membranes, each with its own well-defined microarchitecture (Menon et al., 2019). First, OATP2B1 localization was confirmed in different layers of FMs by IHC (*n* = 3). OATP2B1 expression was localized on the basolateral surface of AECs and on both the apical and basolateral surfaces of CTCs (Figure 1A). In line with previous studies, OATP2B1 was also localized on the basolateral surface of placental syncyiotrophoblasts (Figure 1A). Next, we determined the protein expression of OATP2B1 in FMs by western blotting (*n* = 6). The expression level of OATP2B1 was similar in FMs as in both maternal and fetal sides of the placenta (used as a positive control in our experiment) (Figure 1B). These results show that OATP2B1 location and expression are relatively similar in the FM and placenta.

### Surface Expression of OATP2B1 in FM Cells

**FIGURE 2:**
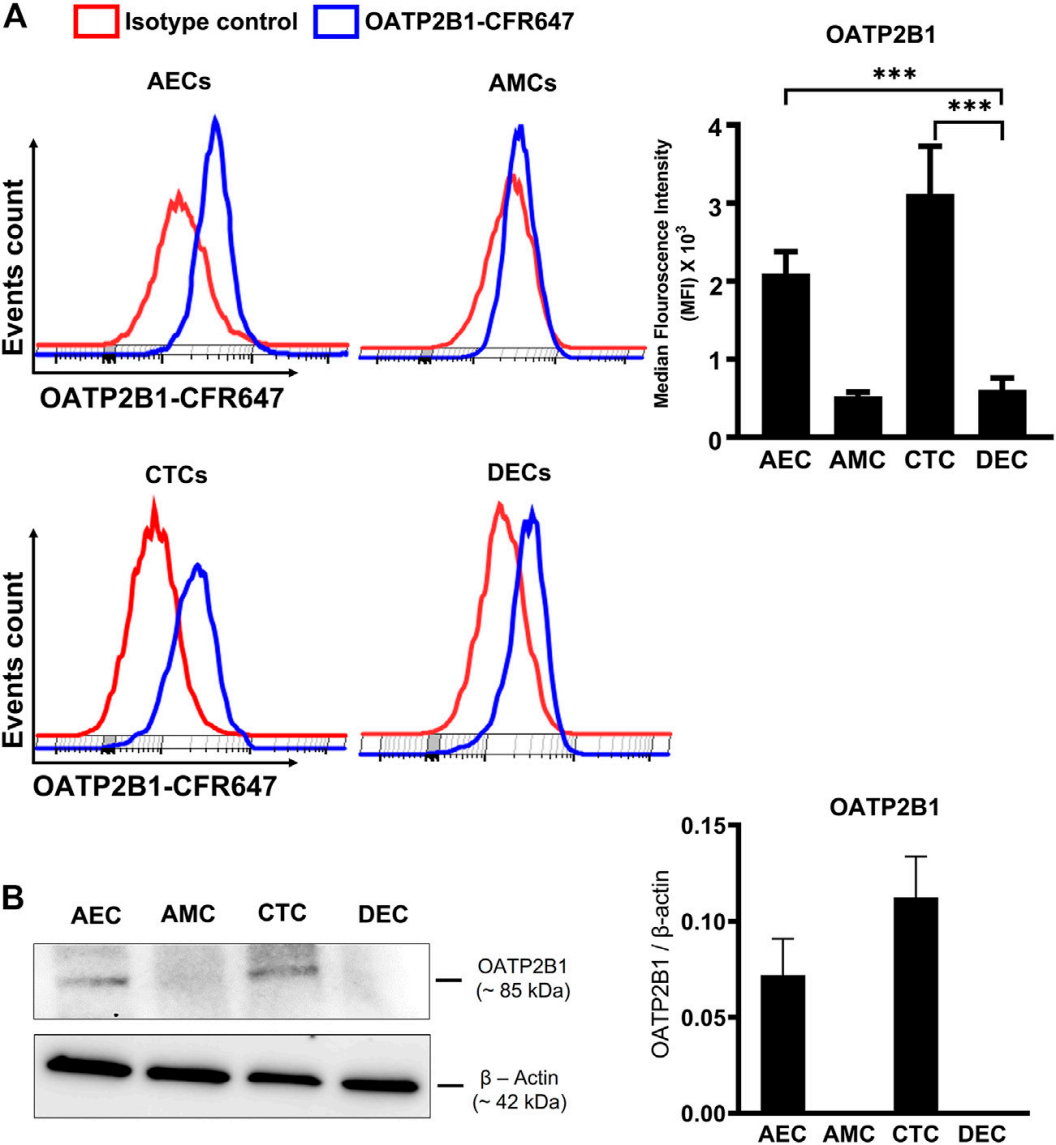
Expression of OATP2B1 transporter protein in human fetal membrane cells. **(A)** Cell surface expression of OATP2B1 in fetal membrane cells (AECs, AMCs, CTCs) and maternal decidual cells (DECs) using primary antibody conjugated with CFR647. Representative histograms of stained samples are shown. The mean fluorescence of these histograms was calculated using the FlowJo Software. **(B)** Western blot analysis and quantification of OATP2B1 in fetal membrane AECs, AMC, CTCs, and maternal DECs. Actin was used as a loading control. Representative blots are shown. Error bars represent mean ± SEM. *n* = 4 biological replicates.

To test whether OATP2B1 is expressed on the surface of fetal membrane cells, flow cytometry analysis was performed on AECs, CTCs, AMCs, and maternal DECs. As shown in Figure 2A, cells expressing OATP2B1 receptor bind to a conjugated antibody and show a distinct shift in flow peak towards right (blue) from the Isotype control stained cells (red). In line with our IHC data, flow cytometry analyses showed differential expression of OATP2B1 on the surface of FM cells (Figure 2A). The surface expression of OATP2B1 on the CTCs was significantly higher compared to the maternal DECs (*p* < 0.5) (Figure 2A). Furthermore, western blot analysis showed OATP2B1 expression in only AECs and CTCs of the FM, but not in AMCs and DECs (Figure 2B).

### Functional Activity of OATP2B1 in FM AECs and CTCs

**FIGURE 3:**
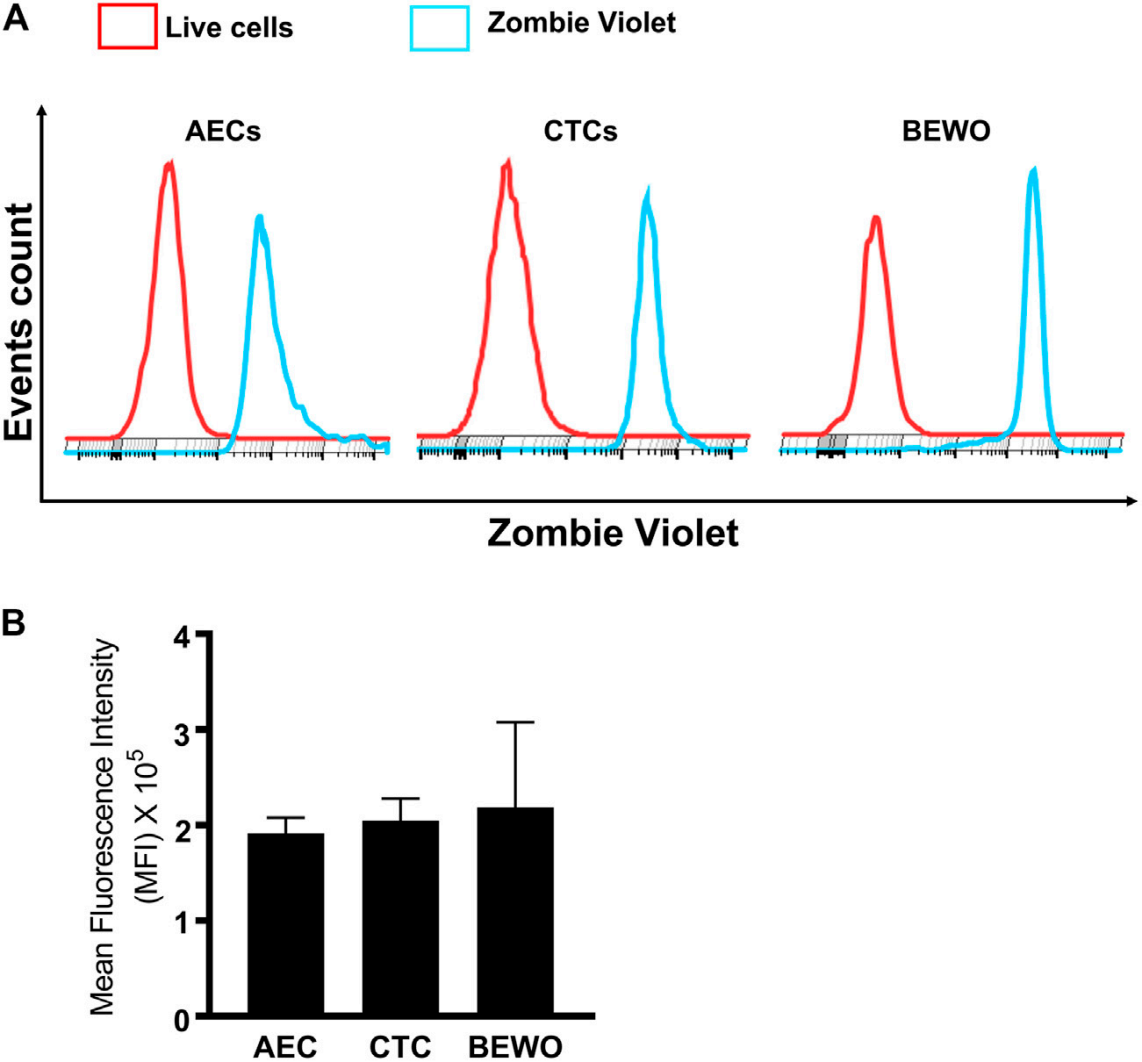
Functional validation of OATP2B1 in fetal membrane and placental cells. **(A)** Functional activity of OATP2B1 in fetal membrane (AECs and CTCs) and placental (BeWo) cells was determined by flow cytometry. Representative histograms of zombie violet dye uptake are shown. **(B)** The mean fluorescence intensity of these histograms was calculated using the FlowJo software. *n* = 4 biological replicates. Error bars represent mean ± SEM.

Next, the zombie violet uptake assay was used to determine the functional role of OATP2B1 in FM cells (Patik et al., 2018; Patik et al., 2015). Zombie violet is a fluorescent viability dye that is taken up by dead cells; however, the presence of OATP can mediate uptake of zombie violet dye in living cells at an extracellular pH of 5.5 (Patik et al., 2018). As an internal control, we have used the propidium iodide (PI) which will stain also dead cells. As shown in Figure 3A, uptake of the zombie violet dye by OATP2B1 receptor showed a distinct shift in flow peak towards right (blue) from the live cells (red). The uptake of zombie violet dye by fetal membrane AECs and CTCs was comparable to the dye uptake observed in the placental BeWo cells (Figures 3A,B). Uptake of the dye by the FM and placental cells suggest that OATP2B1 expressed on cell surfaces of AECs, CTCs and BeWo are functional.

### Oxidative Stress Suppresses OATP2B1 Expression at the Feto-Maternal Interface

**FIGURE 4:**
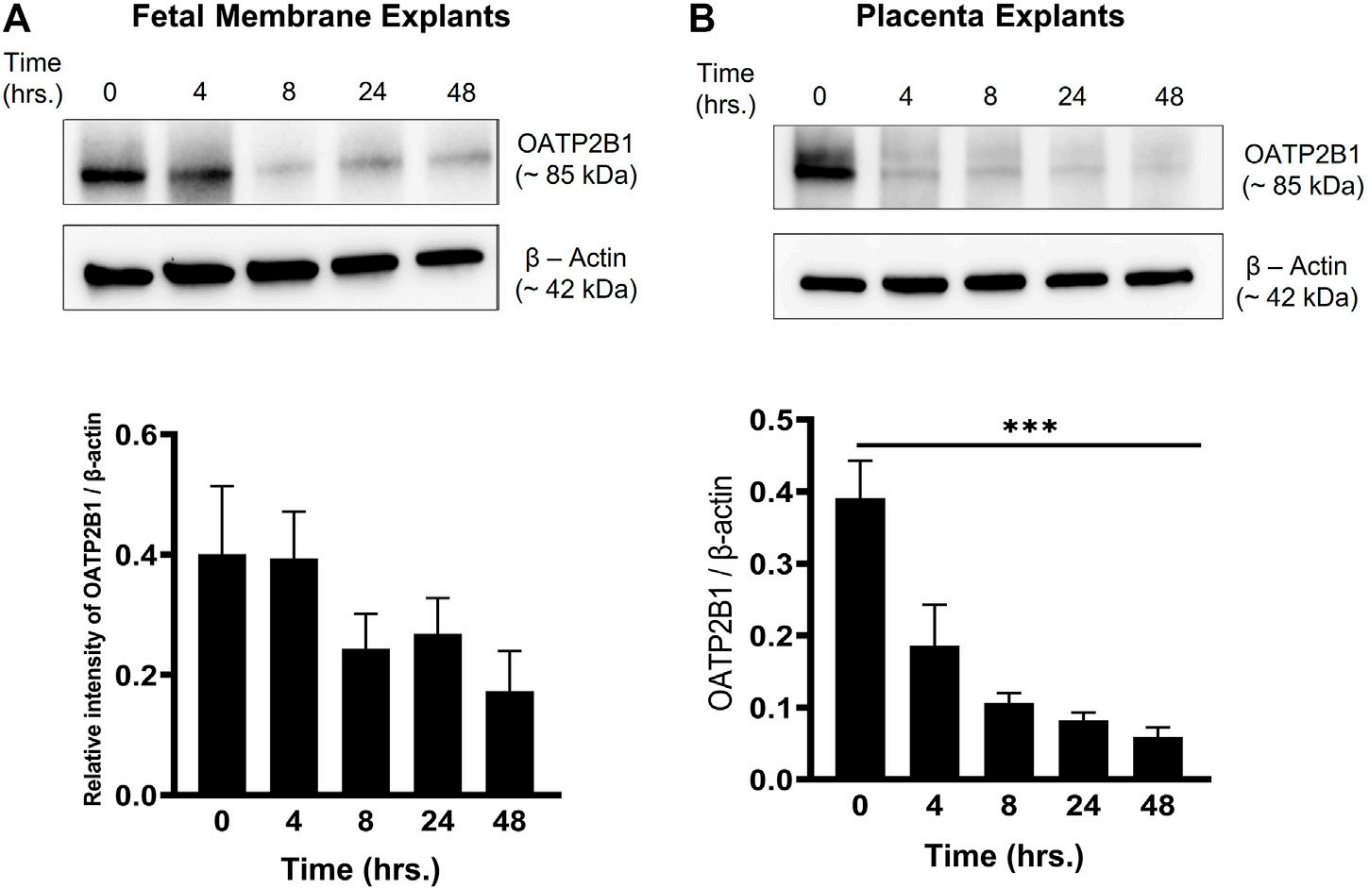
Expression of OATP2B1 in fetal membrane and placenta explants upon stimulation with CSE-induced oxidative stress. **(A)** Western blot analysis and quantification of OATP2B1 in CSE-treated fetal membrane explants shows no significant effect on OATP2B1 expression. **(B)** Western blot analysis and quantification of OATP2B1 in CSE-treated placental explants shows a time-dependent reduction in OATP2B1 expression (***p < 0.001). β-Actin was used as a loading control. Representative blots are shown. *n* = 5 biological replicates. Error bars represent mean ± SEM.

Previous reports show that CSE-induced oxidative stress promotes cellular senescence, cellular transitions, and a pro-inflammatory environment (increased concentrations of pro-inflammatory cytokines) (Polettini et al., 2018; Richardson et al., 2020a). Moreover, a pro-inflammatory environment at the feto-maternal interface has been reported in preterm birth, preeclampsia, and fetal growth restriction (Aouache et al., 2018). Therefore, to determine the effect of CSE-induced oxidative stress on OATP2B1 expression at the feto-maternal interface, FM and placenta explants were exposed to CSE for up to 24 h (0, 4, 8, 24, 48 h) and analyzed for OATP2B1 expression by western blotting. Oxidative stress did not have a significant impact on OATP2B1 expression in the FM (Figure 4A); however, CSE led to a significant decrease in OATP2B1 expression in placental explants (*p* < 0.05) (Figure 4B). These results suggest that oxidative stress or a pro-inflammatory *in utero* environment during pregnancy can lead to differential OATP2B1 expression at the FM-decidua parietalis interface and the placenta-decidua basalis interface.

### Silencing OATP2B1 in CTCs Reduces Drug Transport

**FIGURE 5:**
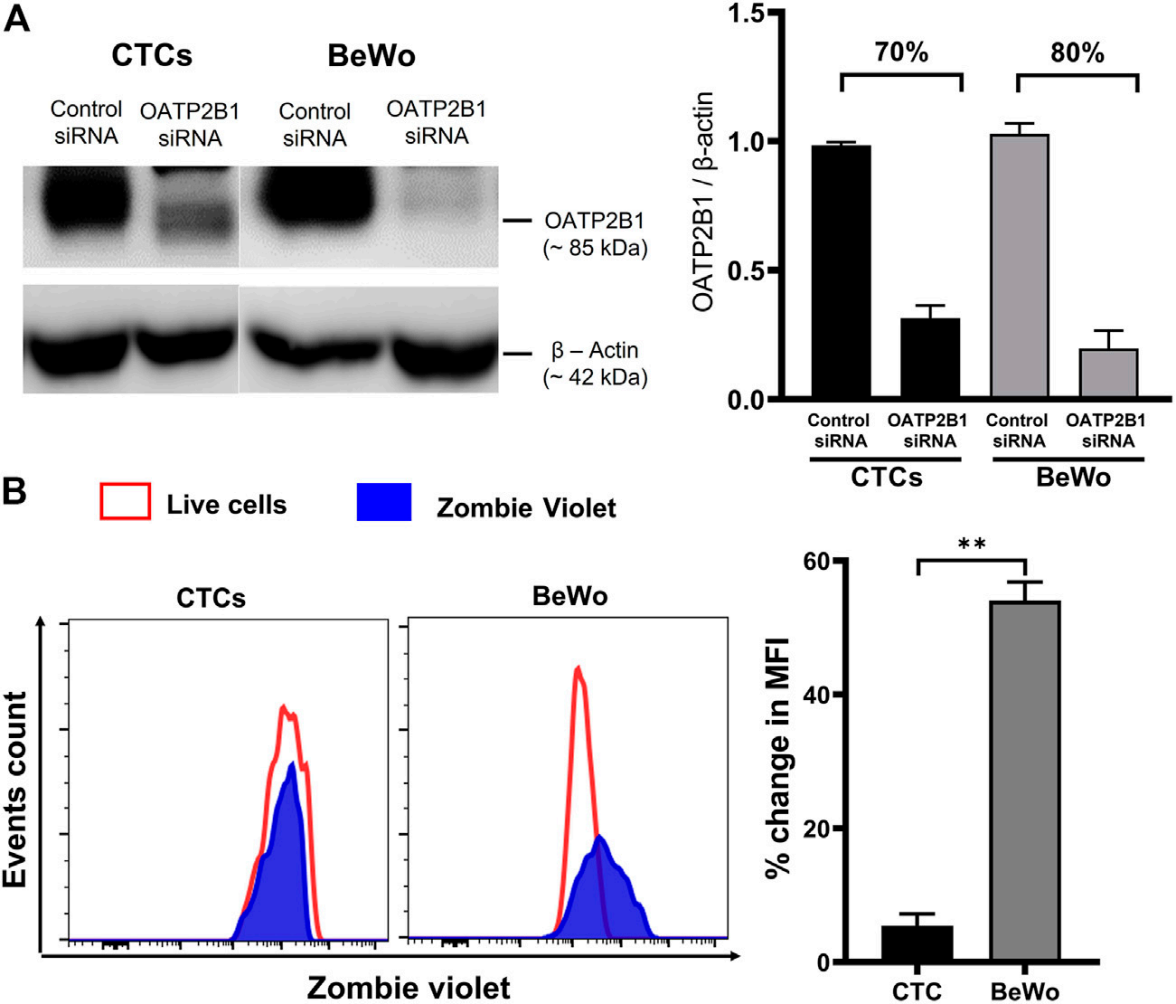
Effect of OATP2B1 silencing on zombie violet dye uptake assay in placental BeWo cells and fetal membrane CTCs. **(A)** Western blot analysis and quantification of OATP2B1 in siRNA-treated BeWo cells and CTCs to determine the efficiency of knockdown. OATP2B1 was silenced in BeWo cells (70%) and CTCs (80%) using short-interfering RNA (siRNA). Non-targeted scrambled siRNA is used as control siRNA. β-Actin was used as a loading control. Representative blots are shown. Error bars represent mean ± SEM. **(B)** OATP2B1 siRNA-treated BeWo cells and CTCs were subjected to the zombie violet dye uptake assay. The data shows that the uptake of zombie violet dye (as indicated by the percent change in mean fluorescence intensity compared to the unstained controls) was reduced to a greater extent in OATP2B1 siRNA-treated CTCs compared to the siRNA treated BeWo cells. Representative histograms of the dye uptake determined by flow cytometry analysis are shown. The percent change in mean fluorescence intensity (MFI) in OATP2B1 knockdown CTCs and BeWo cells (**p < 0.01) compared to their respective controls was calculated using the FlowJo software. *n* = 4 biological replicates. Error bars represent mean ± SEM.

To validate the contribution of OATP2B1 in drug transport, the zombie violet uptake assay was conducted after silencing OATP2B1. A reduction in OATP2B1 expression was determined by western blot analysis in CTCs and BEWO cells (Figure 5A). Knocking down OATP2B1 in CTCs reduced the uptake of zombie violet dye (as indicated by the zombie violet-stained blue peak overlapping with the unstained red peak), suggesting that OATP2B1 in CTCs could play an essential role in the uptake of substrates within the FM (Figure 5B). Interestingly, silencing OATP2B1 had little to no effect on the zombie violet dye uptake in BeWo cells (Figure 5B). We speculate that the differences in uptake results between the FM and placental cells after OXTP2B1 knockdown indicate that possibly in placental BeWo cells other uptake receptors (such as OATP1B1) may also contribute to substrate uptake, whereas in FM CTCs OATP2B1 may play a more predominant role in the uptake of substrates from the maternal DEC.

**FIGURE 6:**
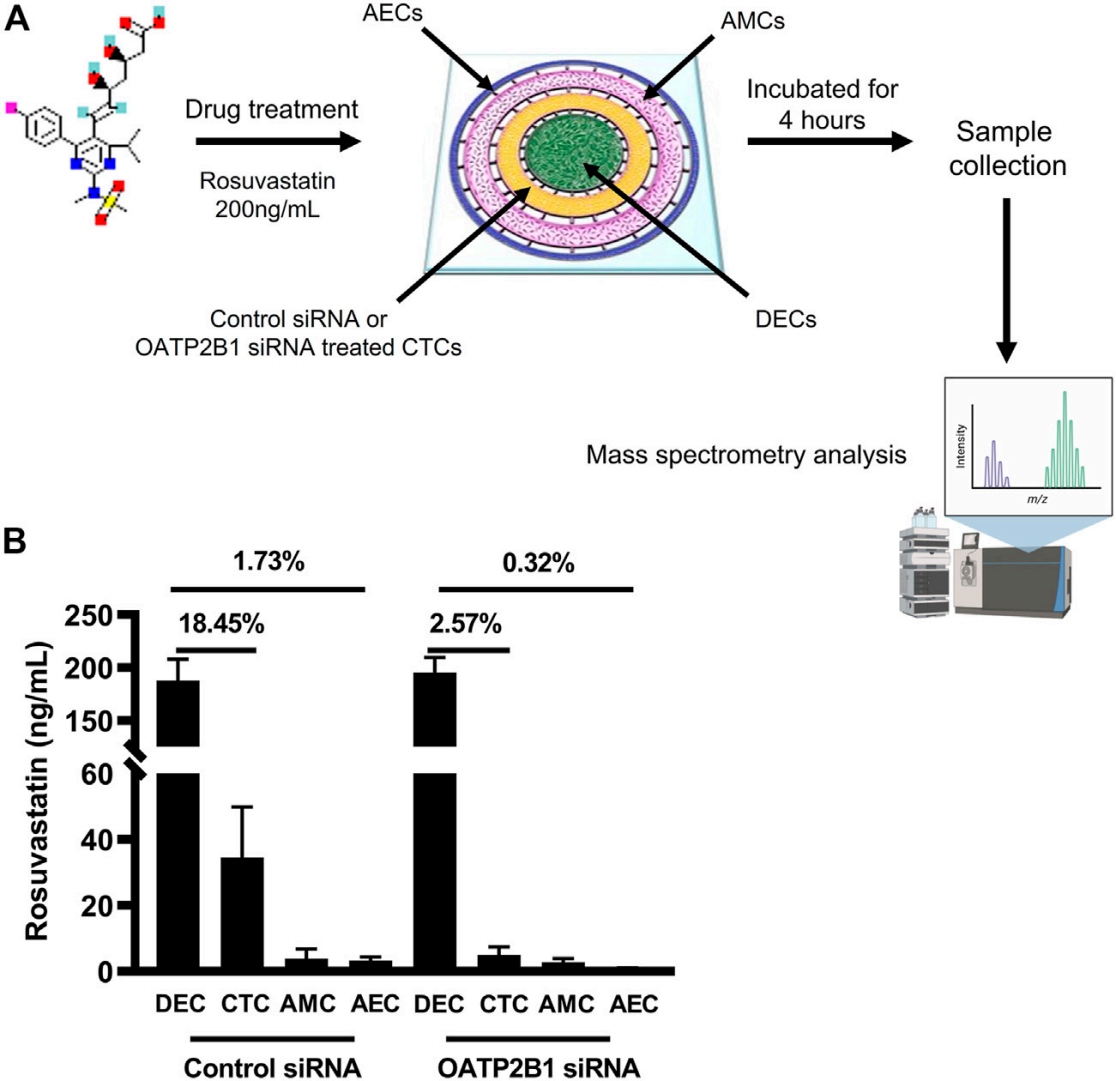
Role of OATP2B1 in rosuvastatin transport across the feto-maternal interface-OOC. **(A)** Schematic showing the four concentric cell culture chambers of the feto-maternal interface-OOC used to determine the role of OATP2B1 in transporting drug across the different fetal membrane chambers [AECs (blue, chamber 1), AMCs (pink, chamber 2), CTCs (yellow, chamber 3), and maternal DECs (green, chamber 4)]. Rosuvastatin (200 ng/ml) was introduced in the decidual chamber of the feto-maternal interface-OOC with either control mock siRNA or OATP2B1-knockdowned CTCs. Following incubation for 4 h, media was collected from different chambers of feto-maternal interface-OOC for mass spectrometry analysis. **(B)** Under control siRNA conditions, 18.45 and 1.73% of rosuvastatin propagated from the maternal DEC to the CTC chamber as well as to the AEC chamber, respectively. OATP2B1 silencing in OATP2B1 resulted in only 2.57% rosuvastatin propagation from the maternal DEC chamber to the CTC chamber, and only 0.32% of rosuvastatin propagation from the DEC chamber to the AEC chamber, and then zero to the CTC chamber. Data is represented as mean ± SEM. Rosuvastatin propagation across the feto-maternal interface -OOC was confirmed from six independent experiments.

To confirm OATP2B1’s role in drug transport, especially in CTCs, the feto-maternal interface-OOC device was used to study the contribution of OATP2B1 in the transport of rosuvastatin through the FM layers. Here, OATP2B1-silenced CTCs were used and compared to normal CTCs. We used stain as the model drug, since they are competitive inhibitors of 3-hydroxy-3-methylglutaryl-coenzyme-A reductase (HMG-CoA reductase) and have been tested in multiple models of pregnancy complications, such as preterm birth and preeclampsia (Istvan, 2003; Basraon et al., 2012). Specifically, we selected rosuvastatin because it is more hydrophilic than other statins (such as pravastatin) and requires protein transporters such as OATP2B1 to enter the cell to inhibit the HMG-CoA reductase enzyme (Climent et al., 2021). This drug was loaded into the DEC chamber (innermost chamber) and its propagation throughout the FM layers measured using mass spectrometry (Figure 6A). Mass spectrometry data suggests that 18.45% of rosuvastatin propagated from the maternal decidual chamber to the fetal CTC chamber under control conditions. siRNA-mediated silencing of OATP2B1 in CTCs however resulted in only 2.57% of rosuvastatin propagating from the DEC chamber to the CTC chamber (Figure 6B). Furthermore, OATP2B1 silencing also reduced rosuvastatin propagation from the DEC chamber to the AEC chamber, where only 0.32% of rosuvastatin was detected in the AEC chamber from OATP2B1 knockdown CTCs compared to 1.73% in non-targeted siRNA control CTCs (Figure 6B). These data further confirm our findings that OATP2B1 could potentially act as one of the most significant drug transport receptors in FM CTCs.

## Discussion

The feto-maternal interfaces are critical for the transport of numerous endogenous substrates, including hormones, steroids, metabolites, neurotransmitters, and exogenous substrates, including drugs (i.e., statins, glycosides, and antivirals) between the mother and the fetus (Menon and Richardson, 2017; Richardson and Menon, 2018). Drug transport across the placental interface is well studied. In contrast, there is a lack of knowledge regarding the contribution of the other feto-maternal interface, namely the FM-decidual interface, as a path for drug transport during pregnancy. The FM provides mechanical, immune, endocrine, transportation, and antimicrobial functions during pregnancy (Menon, 2016). Despite its importance in maintaining pregnancy and fetal growth, research focusing on the role of FM cells in the transport of exogenous and/or endogenous substrates is lacking compared to that of placenta. This is partly because of the avascular nature of FMs, although chorion trophoblast lines with vascular maternal decidua. We report for the first time, to the best of our knowledge, that human FM express functionally intact transporter proteins that can transport drugs. The significance of these findings are: 1) OATP2B1 is expressed in fetal membranes and is mainly localized in the CTC and AEC layers; 2) CTC and AEC cell layers show functionally active cell surface expression of the OATP2B1 transporter; 3) CSE-induced oxidative stress and pro-inflammatory conditions resulted in a time-dependent decrease in the expression of OATP2B1 in the placental explants but not in the FM, and 4) using a four-chambered feto-maternal interface-OOC device, we showed that silencing of OATP2B1 in CTCs significantly reduced drug (rosuvastatin) propagation from the maternal decidua through the CTCs and AMCs to the AECs. In summary, our findings suggest that transporter proteins expressed in the FM-decidual interface can play an important role in drug transport at a similar level, if not more, than the placenta.

OATP2B1 is predominantly involved in the tissue uptake of endogenous (i.e., steroid hormones, metabolites, and neurotransmitters) and exogenous (i.e., statins, glycosides, and antivirals) substrates, and has a sizeable substrate-specific difference in affinity of substrate compared to other OATP transporters because of its multiple substrate binding sites (Ogura et al., 2020). In line with the previous studies, we found that OATP2B1 is expressed on the placental syncyiotrophoblasts basal membrane (fetal-facing). Because of its localization, OATP2B1 plays an important role in drug transport and uptake of sulfates (such as dehydroepiandrosterone; DHEA) from the fetal circulation (Grube et al., 2007). New to the field, OATP2B1 was also identified on the basolateral surface of AECs as well as on the apical and basolateral surfaces of CTCs. Our functional data suggest that OATP2B1 activity on AECs and CTCs is comparable to that seen in placental BeWo cells. Although active OATP2B1 receptors were detected on the surface of FM cells, the transport directionality in each cell types remains to be confirmed, which we plan to conduct as future studies. The presence of OATP2B1 on the surface of AECs and CTCs suggests that fetal membranes can also allow transport and/or diffusion of drugs and their metabolites to reach the intraamniotic cavity, amniotic fluid, and the fetus. Furthermore, the absence of OATP2B1 transporters in the AMCs and maternal decidua denotes different cell-type specificity that may have distinct functions in drug transport mechanisms across fetal membranes. As reported by Richardson et al., AMCs are not necessarily constituents of fetal membranes, but they are transient AECs undergoing a recycling process through cyclic epithelial-to-mesenchymal and mesenchymal-to-epithelial state (Richardson et al., 2020a), a process required to maintain FM integrity. Hence, AMCs are not expected to perform tasks such as transportation of materials between the feto-maternal compartment. Therefore, lack of functional transporters such as OATP2B1 in AMCs are justified.

Interestingly, oxidative stress and inflammation can also affect OATP2B1 expression (Kojovic et al., 2020). For example, a study by Kojovic et al., reported that OATP2B1 expression was increased in placentae obtained from women with preeclampsia (a pregnancy-specific disorder often characterized by increased oxidative stress and increased pro-inflammatory cytokines) (Kojovic et al., 2020). An alteration in the expression of functionally essential transporters (such as OATP2B1) suggests that placental function is compromised during pregnancy complications. However, the impact of oxidative stress and inflammation on OATP2B1 expression in FMs remained unknown. In our study, OATP2B1 expression remained unaffected following CSE treatment, whereas in the placental explants OATP2B1 decreased in a time-dependant manner following CSE treatment. This finding is contrary to previous findings (Kojovic et al., 2020), where OATP2B1 expression was increased in preeclamptic placentae (Kojovic et al., 2020). This could be due to differences between explant phenotypes used, as Kojovic et al., utilized placental tissues obtained from preeclamptic women and here cesarian-derived placental explants were stimulated with CSE. A reduction in the expression of placental OATP2B1 transporter under oxidative stress conditions could potentially lead to a greater risk of substrate accumulation, thus resulting in fetal toxicity. The apparent lack of change of OATP2B1 in fetal membrane explants under similar conditions suggests that fetal membranes can act to transport drugs from fetal circulation under oxidative stress conditions when placental drug transporters may be less functional, providing a new avenue for drug delivery to the fetus.

We further validated the functional role of OATP2B1 in crossing the feto-maternal interface by using an exogenous substrate (rosuvastatin) and siRNA silencing of OATP2B1 in CTCs in our previously developed feto-maternal interface-OOC device. Rosuvastatin is more hydrophilic than other statins (such as pravastatin) and requires protein transporters such as OATP2B1 to enter the cell to inhibit the HMG-CoA reductase enzyme (Climent et al., 2021). Therefore, it is an ideal candidate to use as a substrate for characterizing the functions of transporter proteins. Additionally, rosuvastatin has been reported to reduce oxidative stress-driven inflammation in fetal membranes and has lesser side effects and more effectiveness in regulating inflammatory conditions (Ayad et al., 2018; Kim et al., 2019). Our study found that rosuvastatin propagates from the maternal decidua to the AEC layer of fetal membranes. Interestingly, under control conditions we found substantial rosuvastatin propagation (18.45%) from maternal DEC to CTC chamber, of which only 1.73% of rosuvastatin propagated from CTC to the AEC chamber. We speculated that the marked decrease in rosuvastatin propagation from CTC to AEC chamber as evidenced by reduced rosuvastatin concentration could be due to the fact that we measured only the parent rosuvastatin compound by mass spectrometry, and it is highly plausible that rosuvastatin could be metabolized by the enzymes in the CTC layer. However, if the reduction in rosuvastatin propagation into the AEC chamber could be due to metabolism of rosuvastatin in the chorionic layer remains to be confirmed. Furthermore, siRNA-mediated knockdown of OATP2B1 in CTCs resulted in a significant decrease in rosuvastatin propagation into the CTC chamber and a subsequent reduction into the AEC chamber. Our findings further elucidate the role of human FM in drug transport. Additionally, we have determined that CTCs within the FM, a layer that forms the barrier between mother and the fetus, play a vital role in propagating maternal drugs through the fetal membrane cell layers to reach the amniotic fluid and the fetus.

As noted above, our study provides novel insights into drug transport mechanisms during pregnancy. The discovery of FM as an alternate gatekeeper for drug transport across the feto-maternal interface has broad clinical implications: 1) Designing novel drug delivery systems that could cross not only the placenta, but also FM to improve their pharmacokinetics and therapeutic application; 2) The presence of transport proteins on fetal membranes is expected to expand pharmacologic research during pregnancy, as barrier functions of this second entrance point need to be evaluated thoroughly for currently used and future drugs; The utilization of feto-maternal interface-OOC devices for investigating the role of transporter proteins in drug propagation can be translated to studying drug kinetics during pregnancy and could potentially reduce the time and cost associated with clinical and pre-clinical trials.

Strengths of our study include using an innovative four-chamber microfluidic organ-on-chip device containing different cellular layers of FM and maternal DECs (representing feto-maternal interface) developed and validated in our laboratory. Our feto-maternal interface-OOC can maintain cellular interactions of the amniochorion-decidual interface as seen *in vivo*, mimics the physiological state of feto-maternal interactions during pregnancy, and is fully compatible with various analyses methods such as microscopy and biochemical analyses. Our findings are not without limitations, however. We were limited in testing OATP2B1 substrate (rosuvastatin) propagation only from the maternal decidua through the fetal CTC layer and matrix into the AEC chamber. More importantly, OATPs have differential affinity towards transport substrates, such as steroid sulfates, thyroid hormones, xenobiotics, and drugs; therefore, to fully delineate the functional role of OATP2B1, propagation of various substrates *via* OATP2B1 needs to be further explored. Moreover, substrate propagation from the AEC chamber to the maternal decidua could provide an additional understanding of the transport directionality of OATP2B1s present in AEC and CTC layers of the fetal membranes, which will be explored in future studies.

In summary, we found that FMs can function as a gatekeeper for drug transport. The transporter protein, OATP2B1, expressed on AECs and CTCs, plays an important functional role in drug transportation across the feto-maternal interface. The use of the feto-maternal interface-OOC further confirms, that in addition to the placenta, the FM-decidual interface can transport drugs during pregnancy. Hence, the FM barrier functions need to be evaluated thoroughly for current and future clinical use. This knowledge will improve drug delivery testing during pregnancy and can contribute to designing new drug delivery strategies to better treat adverse pregnancy outcomes.

## Data Availability

The raw data supporting the conclusion of this article will be made available by the authors, without undue reservation.
